# Microstructural Control Strategy Based on Optimizing Laser Powder Bed Fusion for Different Hastelloy X Powder Size

**DOI:** 10.3390/ma15186191

**Published:** 2022-09-06

**Authors:** Jee-Eun Jang, Woosung Kim, Ji-Hyun Sung, Young-Joo Kim, Sung-Hyuk Park, Da-Hye Kim

**Affiliations:** 1 Smart Manufacturing Technology R&D Group, Korea Institute of Industrial Technology, Daegu 42994, Korea; 2 School of Materials Science and Engineering, Kyungpook National University, Daegu 41566, Korea; 3 Development Institute Plant Technology Group, KEPCO KPS Global Institute of Technology, Naju 58326, Korea

**Keywords:** additive manufacturing, laser powder bed fusion, Hastelloy X, microstructure, mechanical property

## Abstract

In additive manufacturing (AM), the powder properties and laser powder bed fusion (LPBF) process parameters influence the quality of materials and building parts. However, the relationship between the size of the powder, LPBF process parameters, and mechanical properties is not well-established. In addition, Hastelloy X (HX) is attracting attention for its excellent high-temperature properties, but it is difficult to process, such as by cutting and milling, because of its high hardness and high ductility. This can be overcome by applying the AM process. We compared the LPBF window process maps for two HX powders of different sizes. Despite their small difference of 19.7% in particle size, it was confirmed that the difference in laser power was more than 40 W, the difference in scan speed was more than 100 mm/s, and the difference in energy density was more than 20% under the optimal process conditions. The as-built specimen had a larger molten-pool size as the energy density was higher, which resulted in the differences in mechanical properties at room temperature and high temperature (816 °C). We considered the control of the size of the powder to obtain the properties required for each temperature condition. The microstructures and mechanical properties of the as-built LPBF specimens were also investigated and compared with those of cast HX. Because of the rapid melting and solidification processes in LPBF, the as-built HX exhibited nano-sized dendrite structures and large internal strain energy. This resulted in the as-built LPBF exhibiting a higher room-temperature tensile strength than the cast material. Under high-temperature conditions, the grain boundary of the as-built LPBF acts as a sliding path, and the as-built LPBF HX showed significantly better high-temperature tensile strength characteristics than the cast HX.

## 1. Introduction

**Figure 1:**
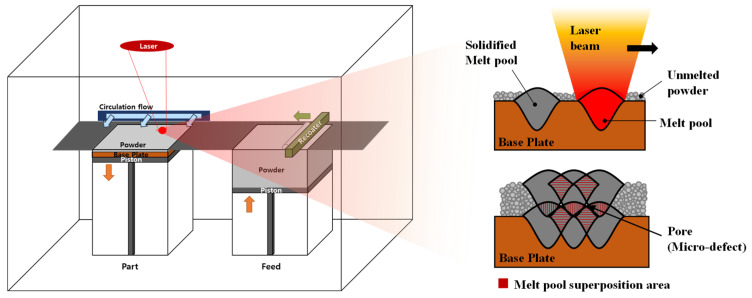
Schematic diagram of laser powder bed fusion (LPBF) process.

The advancements in additive manufacturing (AM) technology have rendered it an attractive option for many industries for the manufacturing of parts that are difficult to manufacture with conventional casting, plasticity, and cutting. Laser powder bed fusion (LPBF) is a metal 3D printing process, which enables the manufacture of complex and sophisticated parts (See Figure 1). It is possible to produce various shapes in a single base plate simultaneously. Therefore, it is very suitable for batch production in a small quantity. Owing to these process characteristics, various industrial fields have tried LPBF for manufacturing [1,2,3].

Many attempts have been made to introduce LPBF to the power generation industry, where swift supply of large quantities of discontinued parts is essential. As the components of a power plant are exposed to extreme high-temperature environments, nickel-based superalloys with excellent high-temperature characteristics are used. Hastelloy X (HX) is a Ni-Cr-Fe-Mo superalloy with high-temperature properties obtained from solid-solution strengthening or precipitation strengthening. The alloy has excellent resistance toward oxidation and air pressure, in addition to its high-temperature properties, making it suitable for components used in the aerospace and power generation industries, such as jet-engine exhausts and turbine blades [4,5,6]. Wrought HX is difficult owing to its high hardness. Therefore, it is primarily worked with casting. However, in recent years, there has been an increasing necessity for fabricating essential parts through AM.

As the LPBF process is being extensively applied in the industry, its process efficiency ought to be investigated for improvement. For this, a high building rate, dimensional accuracy tolerances allowed by the target part, and excellent surface roughness are required during the fabrication of parts through LPBF [6,7,8]. Parts fabricated by metal 3D printing have larger inward features and smaller outward features as the parts shrink because of the rapid cooling process. S. Giganto et al.’s [9] research shows that the dimensional error caused by these causes is maximized on a sloped surface (especially on a 45° one), so one should be careful. The LPBF process involves fabrication by melting metal powder with a laser heat source and possesses numerous control process parameters [1,3,8,10,11,12]. Among them, laser beam power and laser scanning speed have been observed to significantly influence the quality and process efficiency [7,10,13]. Furthermore, the particle shape, size, and composition of the powder material significantly influence melting through the laser heat source. These factors are directly related to the quality of the AM-fabricated material. Therefore, it is necessary to select the optimal process conditions by controlling the primary LPBF process parameters and powder properties [14,15,16,17,18]. Due to the importance of process optimization and the difficulty of controlling many variables, recent studies such as the selection of optimal process variables using machine learning [19] and the optimization of properties through post-heat treatment [20] are being actively conducted.

The microstructure of the materials fabricated through LPBF and casting are different: in contrast to casting, the LPBF process grows only as a primary dendrite because the material melts/solidifies rapidly, and the secondary dendrite growth time and grain growth time are insufficient. The dendrite resides in a molten pool that forms as the laser melts the powder [21,22]. The unique microstructure of these materials fabricated by LPBF is a factor influencing the physical properties that are different from those of the cast material.

This study analyzes the influence of HX powder size on the mechanical properties of parts fabricated by LPBF, for the selection of optimal LPBF process parameters. The microstructure and mechanical properties of HX, fabricated by casting and LPBF, were comparatively analyzed. Forty-eight specimens were fabricated with two types of HX powder of different sizes and subjected to a laser operating at 120–260 W and 500–1000 mm/s scanning speed under different conditions. The fabricated specimens were organized as a process window map based on the density measurement.

## 2. Materials and Experimental Procedures

### 2.1. Material

#### Hastelloy X Powder Size Distribution and Chemical Composition

**Figure 2:**
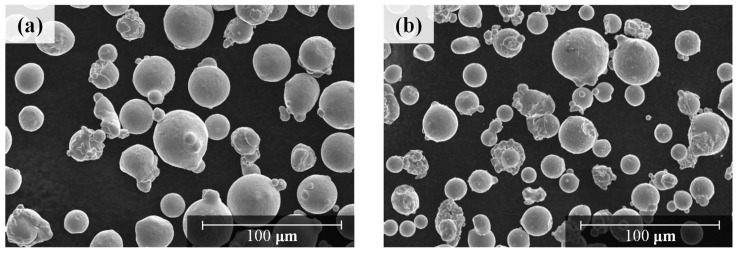
Shape image of two types of Hastelloy X powder used in the LPBF process: (**a**) Powder I, (**b**) Powder II.

**Figure 3:**
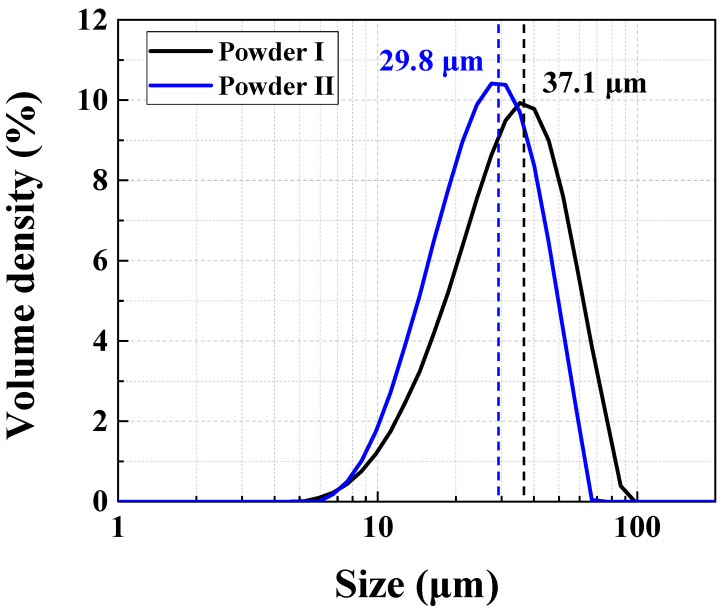
Particle size distribution of FS GH3536 (Powder I) and nickel alloy HX (Powder II).

Two types of Hastelloy X powders were selected for this study. The powder composition was analyzed using energy dispersive X-ray spectroscopy (EDS) (X-Max 50 mm, HORIBA, Japan), gas chromatography, and C/S determinator. The alloys FS GH3536 (Powder I) and nickel alloy HX (Powder II) gas-atomized powders, provided by Farsoon Technologies and EOS GmbH, respectively, were selected as they are widely used in AM technologies. The composition of Powder I is 16.1% Cr-16.0%Fe-8.5%Mo-0.7%Co-0.5%W-0.02%Si-0.2%Al-bal. Ni and trace amounts of Ti, Nb, O, N, C, and S. The composition of Powder II is 22.8% Cr-18.8%Fe-8.6%Mo-1.7%Co-1.0%W-0.1%Si-0.5%Al-bal. Ni and trace amounts of Ti, Nb, O, N, C, and S. Figure 2 shows that the morphologies of the powders that were unused were usually spherical. In this experiment, as the microstructures of the AM specimens varied according to the particle size of the powder, the particle size distribution of the two types of Hastelloy X powders were analyzed using a field emission scanning electron microscope (SEM) (SU8020, Hitachi, Japan) and laser granulometry (Mastersizer 3000 wet dispersion units, Malvern, UK). The results of the analysis showed that the average particle size D [3,4], which means the average value of the powder volume in the overall measurement result, of Powder I and Powder II were 37.1 and 29.8 µm, respectively. That is, Powder I exhibited a larger powder distribution than Powder II, as shown in Figure 3.

### 2.2. Experimental Procedures

#### 2.2.1. Specimens Prepared by Powder Bed Fusion Method

Hastelloy X cuboid specimens (10 × 10 × 20 mm) and cylindrical specimens (diameter: 12.3 mm, length: 85 mm) were fabricated with Farsoon 271 M (Farsoon Technologies, Changsha, China). Tensile test specimens were fabricated by vertical and horizontal building. This system used a Yb-fiber laser rated at 500 W. The scanning was performed at a base plate temperature of 150 °C under a nitrogen gas atmosphere. The main parameters applied in the LPBF process are:

- Laser beam power: 120–260 W,
- Layer thickness: 30 μm,
- Laser scanning speed: 100–500 mm/s,
- Laser scanline spacing: 115 μm.

#### 2.2.2. Microstructure Observation

The as-built and cast specimens were intermediately polished using 320–2000 grit SiC paper to compare their cross-sectional porosity, microstructure, and texture. The specimens were final polished using 3 and 1 μm diamond suspension and finished with colloidal silica of particle size 0.04 μm. The specimens were then etched electrolytically with Kalling’s No. 2 reagent (5 g CuCl_2_ in 100 mL HCl + 100 mL CH_3_CH_2_OH), at 6.0 V, for 5 s. For the measurement of cross-sectional porosity, cuboid specimens were cut vertically and horizontally.

The pore shape and size of the cross-sections were observed with an optical microscope (OM) (Axio Observer A1m, Carl Zeiss, Jena, Germany), and the porosity values were calculated from the images of the cross-sectional pores using IMT i-Solution Inc. software.

The etched specimens were investigated for microstructure characterization and the precipitation size and distribution using OM and SEM. In addition, EDS and X-ray diffraction (XRD) analyses were performed for the phase analysis of precipitates and matrix identification through microstructure analysis. Electron backscatter diffraction (EBSD) was used for texture analysis, and the EBSD data were analyzed using Oxford instrument’s Aztec version 4.3 software (AztecCrystal 4.3, Oxford instruments, High Wycombe, UK).

#### 2.2.3. Tests for Mechanical Properties

Specimens fabricated, subject to various process parameters, were analyzed for density and cross-sectional porosity to select the optimal process parameters. A helium gas pycnometer (AccuPyc II 1340, Micromeritics, Norcross, GA, USA) was used to measure the density of the fabricated specimens. The hardness was measured at 5 points using the Vickers hardness test machine (Wilson VH3300, Buehler, Lake Bluff, IL, USA) under a constant press load of 19.61 N (HV2). The as-built and cast specimens adhering to the ASTM E8 standard were subjected to a room temperature (24 °C) tensile test under a strain rate of 8.0 × 10^–4^ s^−1^. In addition, a high-temperature (816 °C) tensile test was performed under a strain rate of 8.3 × 10^−4^ s^−1^. The creep rupture test was conducted in accordance with the ASTM E139-11 test standard at 816 °C, 103 MPa load, and Ar gas environment with a specimen manufactured according to the ASTM E8 standard.

## 3. Results and Discussion

### 3.1. Laser Powder Bed Fusion (LPBF) Process Optimization

**Figure 4:**
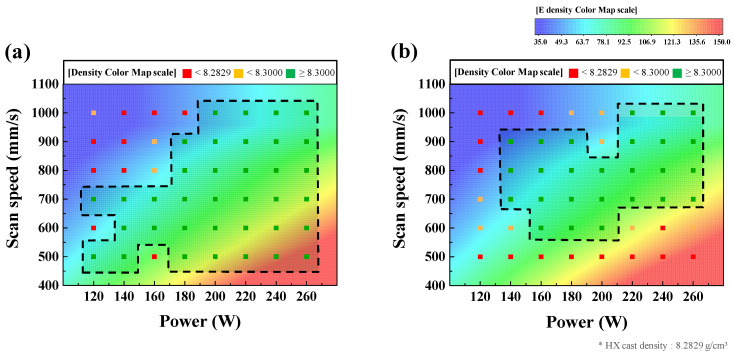
Process window map for (**a**) Powder I specimens and (**b**) Powder II specimens fabricated at laser powers of 120–260 W in increments of 20 W, and scan speeds of 500–1000 mm/s in increments of 100 mm/s. Red points indicate specimens with a density lower than that of Hastelloy X cast, yellow points indicate specimens with a density higher than that of Hastelloy X cast but less than 8.3000 g/cm^3^, and green points indicate specimens with a higher density than 8.3000 g/cm^3^.

In the LPBF process parameter optimization test, the laser scan line spacing was constant, while the laser beam power and scanning speed were varied for process window mapping. Figure 4 shows the overlapped process window map and energy density color map to improve the clarity of the process window area setting [23,24]. Energy density is used to compare the energy used for LPBF by combining some process parameters. Energy density is the value obtained by dividing the laser beam power used by the laser scanning speed, the laser beam diameter, and the powder layer thickness. In the process window map, specimens of density <8.2829 g/cm^3^, which is the density of HX cast, are shown as red points, and those with greater density are shown in orange and green points. Specimens measured at a density higher than 8.3000 g/cm^3^ were marked with green points and were grouped within a dotted line to classify the area as a ‘stable building zone’. Figure 4 shows that the stable building zone in the process window map of Powder I exists in the lower right corner in contrast to that of Powder II. The average energy density of the stable building zone of Powder I (86.5 J/mm^3^) > Powder II (58.0 J/mm^3^). This was caused by the large powder size, requiring greater energy for melting than for smaller particle size. The optimal process parameters that satisfy the following conditions within the stable building zone were selected:

- High density and low surface porosity: strong mechanical properties by reducing internal defects,
- Low surface roughness: high dimensional accuracy of specimens.

For Powder I and II, the optimal laser beam power was 260 and 160 W, respectively; correspondingly, the optimal laser scanning speeds were 900 and 800 mm/s, respectively.

**Figure 5:**
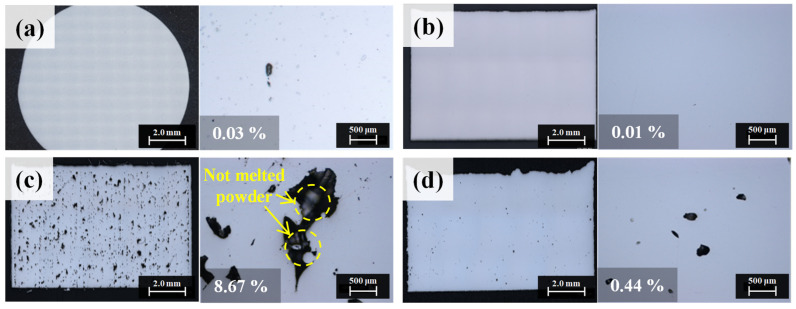
(**a**) Cross-sectional porosity result of Hastelloy X cast specimen, cross-sectional porosity results of laser power bed fusion (LPBF) process window map: (**b**) void-free cross section in the stable region, (**c**) lack of fusion defects primarily occurring in the low energy density region, (**d**) keyhole defects primarily occurring in the high energy density region.

After the as-built specimen was fabricated by applying the optimal process parameters, the vertical and horizontal cross-sectional porosities of the specimen were detected through the OM (See Figure 5b). The cross-sectional porosity of the cast in Figure 5a was 0.03%, whereas that of the stable building specimen was 0.01%. The cross-sectional porosity, maximum pore size, and density of the as-built specimens for each powder are summarized in Table 1. The primarily observed pores were small and spherical, with an average size of 3–5 μm. These pores are observed to be metallurgical pores formed by the trapped gas in the molten pool between the agglomeration of the melted powder. On the other hand, the process window map in Figure 4 shows that when low power and high scan speed or high power and low scan speed were applied to the process, the density of the specimens decreased. Under low power and high scan speed, the energy density applied to the process was insufficient to melt all the powder. Therefore, the melted area was irregular and resulted in large pores and low density, as shown in Figure 5c. On the other hand, under high power and low scan speed, powder spattering occurred in the direction of laser scanning, as the energy density was higher than necessary [14]. This causes a morphological problem, where the outer part of the specimen rises higher than the center of the specimen [25]. In addition, spattered powders fall on the layer, causing surface defects, such as balling, and the reduction in the quality of fabrication [14,25,26]. In addition, the greater the amount of heat input because of the high laser beam power, the more heat is induced on the surface of the molten pool, and as the temperature increases, the laser reflectance decreases, resulting in a deeper molten pool [27]. The gas trapped at the bottom of the deep molten pool remains trapped, and the surrounding metal solidifies to form a circular void resembling a keyhole void, as shown in Figure 5d.

### 3.2. Microstructure of As-Built and Cast Hastelloy X Alloy

**Figure 6:**
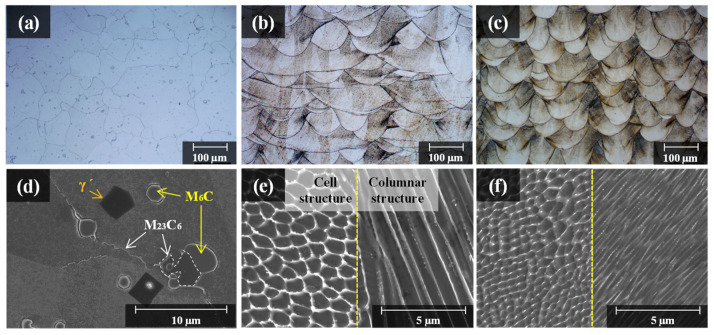
OM micrographs of the (**a**) cast Hastelloy X specimens and fabricated Hastelloy X specimens with different process parameters; (**b**) Powder I as-built specimen; (**c**) Powder II as-built specimen; SEM micrographs of (**d**) cast, (**e**) Powder I as-built specimen, and (**f**) Powder II as-built specimen.

After detecting the cross-sectional pores, the specimen was etched to observe the molten-pool morphologies. The as-built LPBF possessed a unique microstructure significantly different from that of the general cast. The cast solidifies relatively slowly, resulting in insufficient time for grain growth. Therefore, generally, they form into hexagonal grains as shown in Figure 6a,d. On the other hand, the as-built LPBF does not have enough time for grain growth, owing to the rapid melting and solidification processes. Therefore, a molten pool composed of dendrites is formed, as shown in Figure 6e,f. In addition, as its rate of solidification is high, there is insufficient time for secondary dendrites to grow. Therefore, only primary dendrites grow. This primary dendrite can be observed as a circular cell structure when cut vertically in the growth direction and as an elongated rod-shaped columnar structure when cut horizontally in the growth direction.

**Figure 7:**
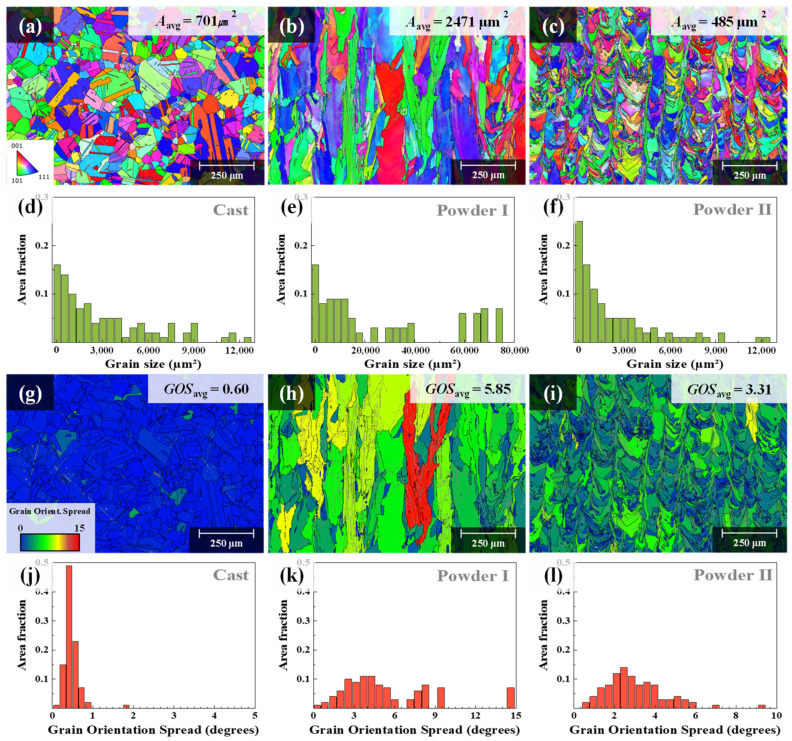
Electron backscatter diffraction (EBSD), inverse pole figure (IPF) and grain boundary (GB) images of building direction (vertical) plane, and the distribution of grain sizes (**a**,**d**) cast, (**b**,**e**) Powder I as-built, and (**c**,**f**) Powder II as-built; grain orientation spread (GOS) map and the distribution of GOS values of (**g**,**j**) cast, (**h**,**k**) Powder I as-built, and (**i**,**l**) Powder II as-built.

Figure 6b,c show the OM image of the molten pool of the as-built specimen; Figure 6e,f show the SEM image of the micro cell/columnar structure inside the molten pool. The size of the molten pool and cell/columnar structures decrease relatively, with the decrease in the energy density applied to the LPBF process. A decrease in the size of the molten pool and cell/columnar structures indicates an increase in their boundary area. The grain boundary of the cell/columnar structure allows micro-defects such as voids or cracks. Therefore, a greater number of pores were observed in the Powder II as-built specimen than the other specimens, as shown in Table 1, as it possessed larger cell/columnar structure and grain boundary area. The difference in the molten-pool size can be compared with clarity through the overlapped images of the inverse pole figure (IPF) map showing the microstructure orientation and the grain boundary (GB) map showing the grain boundary (See Figure 7a–f). The comparison of grain sizes is as follows: Powder II as-built (485 μm^2^) < cast (701 μm^2^) < that of Powder I as-built (2471 μm^2^). Figure 7g–l is a grain orientation spread (GOS) map showing the mean value of the grain’s internal orientation deviation and a graph of the measured GOS value. In contrast, owing to the relatively slow solidification process, the dislocation density and internal strain energy decreased as dislocations were arranged. Therefore, the measured GOS_avg_ value was low (0.60°). On the other hand, in the case of the LPBF as-built specimen, a high-temperature gradient generated from the rapid melting and solidification processes caused material deformation and the accumulation of high internal strain energy. Therefore, the GOS_avg_ for the Powder I as-built and Powder II as-built specimens were 5.85 and 3.31, respectively. In the case of the LPBF as-built specimens, the Powder I as-built specimen fabricated under a high beam power exhibited a higher GOS_avg_ value than the Powder II as-built. For the high laser beam power, the volume of the molten pool and grain were further increased, and a relatively large temperature gradient was generated in the molten pool. This resulted in a high residual stress and high strain distribution.

### 3.3. Mechanical Property Investigation of As-Built and Cast Hastelloy X Alloy

**Figure 8:**
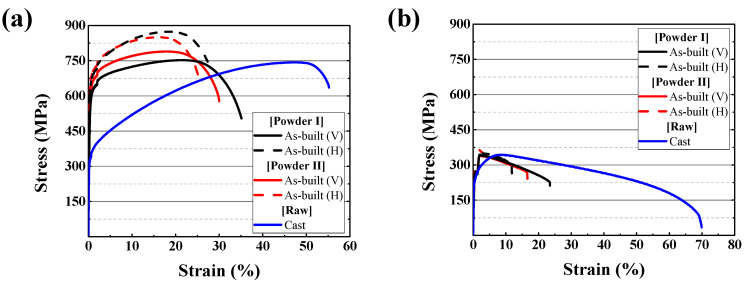
Tensile test stress–strain relationship results at (**a**) room temperature and (**b**) high temperature (816 °C).

**Figure 9:**
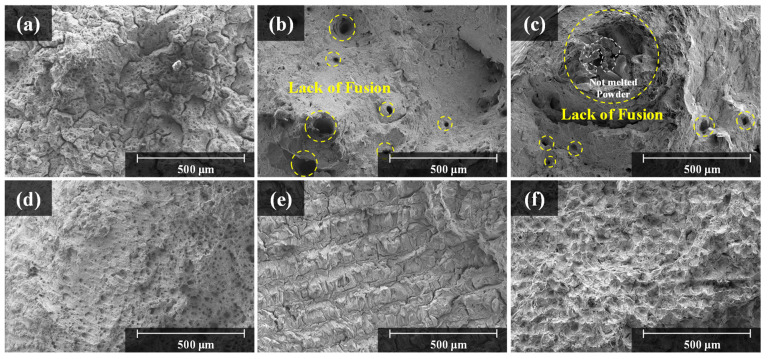
Tensile fracture surface characteristics of the cast and fabricated Hastelloy X specimens with different process parameters: (**a**) cast, (**b**) Powder I as-built, (**c**) Powder II as-built specimens; high-temperature (816 °C) tensile fracture surface characteristics of (**d**) cast, (**e**) Powder I as-built and (**f**) Powder II as-built specimens.

Figure 8 shows the results of the room and high-temperature tensile strength tests of the LPBF as-built and cast specimens. Figure 8a shows that the LPBF as-built specimen exhibits higher tensile strength than the cast specimen at room temperature. The sub-grain boundary and the high internal strain energy of the LPBF as-built specimen act as strain hardening factors to strengthen the material. As the gliding of the dislocation becomes difficult because of that reinforcing mechanism, the tensile strength of the as-built increases, but the elongation is lower than that of the cast. Unlike the room-temperature tensile strength, where dislocation movement is a significant factor resulting in material fracture, at high temperatures, the grain boundary sliding (GBS) acts as the major fracture factor owing to the mobility of atoms near the grain boundary [28,29,30]. Therefore, the larger the grain size, the greater the high-temperature strength. The fracture caused from such a GBS can be clearly confirmed through fracture surface analysis. In the case of the LPBF as-built specimen (Figure 9e,f), the fracture occurred along the grain boundary, which was the primary propagation region of GBS. Owing to this inverse Hall–Petch effect, it can be observed from the graph in Figure 8b that the Powder I as-built specimen with the largest grain size exhibits the best high-temperature strength. In the case of the Powder II as-built specimen, despite having a smaller grain size than that of the cast it exhibits a higher high-temperature strength than cast because of the high internal strain energy. As the high density of dislocations accumulates at the molten-pool boundary, which is a factor for high internal strain energy in the material, the dislocations act as obstacles to GBS.

The mechanical properties observed from the directions of the vertical and horizontal stacks are different in the LPBF as-built. The stacked horizontal LPBF as-built specimen (H) has higher strength and lower elongation than the stacked vertical LPBF as-built specimen (V). The strength of the stacked vertical LPBF as-built specimen (V) is reduced because the laser scanning path acts as a crack propagation path. On the other hand, the strength of the stacked horizontal LPBF as-built specimen (H) was improved because the laser scanning path hinders crack propagation [31,32].

The LPBF Powder II as-built specimen with a relatively small grain size exhibits a high room-temperature tensile strength according to the Hall–Petch effect. On the other hand, at high temperatures, the Powder I as-built specimen, which has a larger grain size, exhibited a better high-temperature strength than the Powder II as-built specimen because of the inverse Hall–Petch effect [33].

### 3.4. Creep Rupture Time of Hastelloy X Alloy LPBF as-Built Specimen

**Figure 10:**
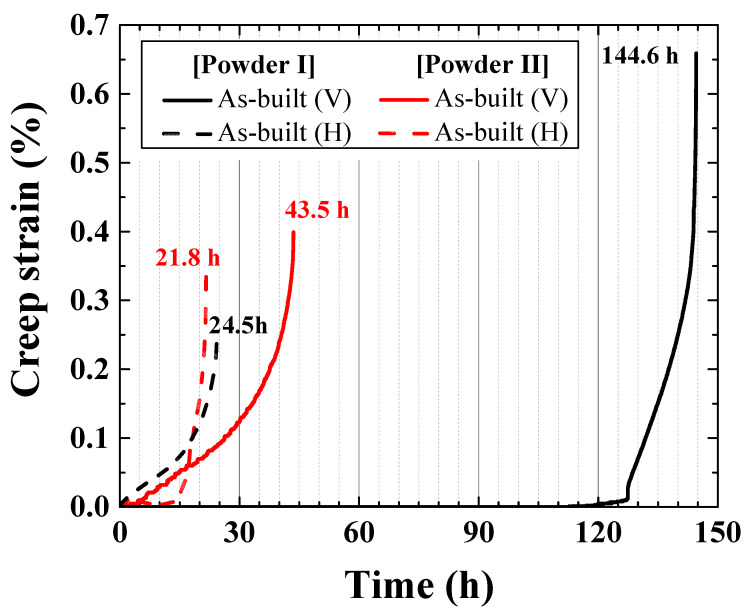
Powder I and II as-built creep test results and creep rupture time change comparison. Powder I is indicated by black lines, and Powder II is indicated by red lines. The vertical (V) and horizontal (H) as-built specimens are indicated by solid lines and dotted lines, respectively.

**Figure 11:**
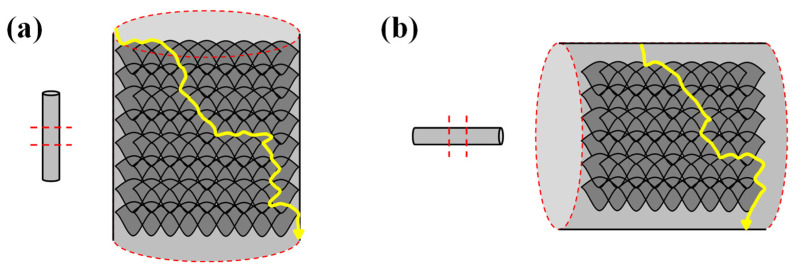
Schematic illustration of the difference of creep crack propagation path according to the building direction: (**a**) stacked vertical as-built; (**b**) stacked horizontal LPBF as-built.

**Figure 12:**
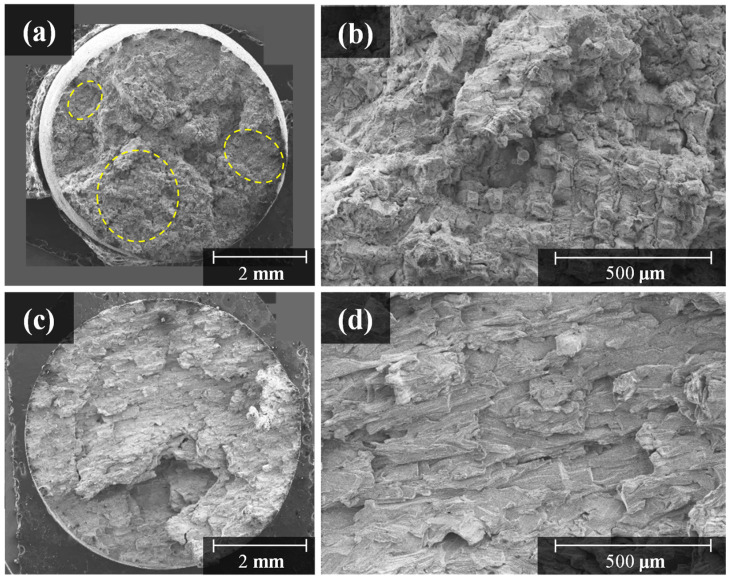
Creep fracture surface characteristics of Hastelloy X specimens fabricated with different deposited direction: (**a**,**b**) are stacked vertical LPBF as-built specimens; (**c**,**d**) are stacked horizontal LPBF as-built specimens.

The creep property of Hastelloy X fabricated through LPBF differed depending on the deposition direction and the size of the powder. As can be observed from the creep rupture graph in Figure 10, the as-built (V) deposited in the vertical direction exhibits a better creep rupture life than the as-built (H) deposited in the horizontal direction. Furthermore, the Powder I as-built using a relatively large powder has a better creep rupture life than the Powder II as-built. These effects are caused by the grain size and depth of penetration between the molten pools. For high-temperature tensile strength, the GBS is a major cause of creep rupture. However, in the case of the creep test, since the downward stress, which is lower than that of the tensile strength test, is applied downward for a long time, fracture occurs at a very slow rate. Figure 11 shows the case of creep rupture, where the cracks gradually propagate downward along the molten-pool boundary. The as-built (V) in Figure 11a possesses a greater number of layers than the as-built (H) in Figure 11b; the molten-pool boundary area where GBS is generated is wider. Therefore, the as-built (V) exhibited a better creep rupture life than the as-built (H). In the same way, since the Powder I as-built specimen exhibits a deeper penetration between molten pools and a more irregular molten-pool boundary than Powder II as-built specimen, it inhibits GBS and results in a higher creep rupture life (Figure 7b,c). However, the creep rupture life of the as-built (H) with similar number of layers resembles those of Powder I and II. The creep fracture surface image in Figure 12 shows that the rupture occurs along the molten-pool boundary in Figure 12a, and a scanning line pattern is observed in some areas of the fracture surface.

## 4. Conclusions

In this study, we investigated the effect of powder size on the mechanical properties of specimens for the optimization of AM process parameters. The differences in the microstructure and mechanical properties of the LPBF as-built and cast specimens were compared and analyzed.

(1) To investigate the LPBF optimization process according to the powder size, two types of Hastelloy X powders with different sizes were used by varying the laser power and scanning speed. The specimens were then fabricated under various LPBF process parameters and the densities of all specimens were measured, which were organized into a process window map. According to the process window mapping result, with increasing powder size, a stable building zone existed in the lower right corner, which is a region with a large energy density. This is because a large quantity of energy is required to melt a large powder.

(2) Powder II, which has a relatively low melting energy, exhibited a smaller molten-pool size than Powder I. According to the Hall–Petch effect, the Powder II as-built with a large grain boundary area that prevents dislocation movement has a higher room-temperature strength. On the other hand, at high temperatures, since the GBS is the primary factor of fracture, the Powder I as-built with a small grain boundary area has an excellent high-temperature strength according to the inverse Hall–Petch effect.

(3) The LPBF as-built is strain-hardened, owing to the cell/columnar structure sub-grain boundary and high internal strain energy, which was formed by the rapid melting and solidification processes; therefore, it has better strength than cast.

(4) Comparing the mechanical properties according to the ‘building direction’ of the tensile specimen, the horizontally stacked specimens were superior to the vertically stacked specimens at both room and high-temperature tensile strength tests. This mechanical anisotropy according to the building direction may be caused by the laser scanning path, which is a trace generated by the passing of the laser.

(5) The creep rupture life is also affected by the deposition direction and the molten-pool size. The as-built (V), which was perpendicular to the deposited direction and the stress application direction in the creep test, exhibited a better creep rupture life than the as-built (H). In addition, since the size of the powder is large, Powder I with a large molten-pool size exhibited a better creep rupture life than Powder II.

(6) In the case of obtaining excellent high-temperature properties with the LPBF process, when a large powder is used, a stable LPBF process is possible even at high energy density, and it is simultaneously possible to form large molten pools and grains to obtain excellent high-temperature properties.

## Figures and Tables

**Table 1:** The density of Powder I and Powder II corresponding to as-built and cast samples measured by gas pycnometer, and details of pore observation results on the sample cross-cut section: cross-sectional porosity, measured maximum pore area, average pore area, and tracked pore numbers.

	Cast	As-Built	
		Powder I	Powder II
Density (g/cm^3^)	8.28	8.38	8.34
Cross-Section Porosity (%)	0.03	0.01	0.01
Maximum Pore Area (μm^2^)	3084.13	682.56	764.07
Average Pore Area (μm^2^)	162.35	53.68	32.13
Tracked Pore Numbers	194	208	344

## Data Availability

Not applicable.
